# Epigallocatechin-3-gallate Can Prevent Type 2 Human Papillomavirus *E7* from Suppressing Interferon-Stimulated Genes

**DOI:** 10.3390/ijms22052418

**Published:** 2021-02-28

**Authors:** Ji Young Song, Ju Hee Han, Yumee Song, Ji Hyun Lee, Soon Yong Choi, Young Min Park

**Affiliations:** 1 Program of Immunology & Microbiology, Department of Biomedicine & Health Sciences, Graduate School, The Catholic University of Korea, 222, Banpo-daero, Seocho-gu, Seoul 06591, Korea; sjy8699@gmail.com; 2 Department of Dermatology, Seoul St. Mary’s Hospital, College of Medicine, The Catholic University of Korea, 222, Banpo-daero, Seocho-gu, Seoul 06591, Korea; alwaysmine8@gmail.com (J.H.H.); yumeesong@naver.com (Y.S.); yiji1@hanmail.net (J.H.L.); 3 Department of Biological Science and Biotechnology, College of Life Science and Nano Technology, Hannam University, 1646, Yuseong-daero, Yuseong-gu, Daejeon 34054, Korea; sychoi@hnu.kr

**Keywords:** *E7*, epigallocatechin-3-gallate, human papillomavirus, interferon-stimulated genes, type I IFN signaling pathway

## Abstract

Human papillomavirus (HPV) in high-risk groups is known to suppress the type I interferon (IFN) signaling pathway leading to the transcription of interferon-stimulated genes (ISGs), which have many antiviral functions. However, the effects of HPV on the action of various ISGs in low-risk groups are not fully understood. We aimed to investigate whether antiviral ISGs are expressed in transfected keratinocytes with type 2 HPV (HPV-2) *E7*. The mRNA and protein expressions of ISGs and type I IFN signaling pathway components were evaluated by quantitative real-time polymerase chain reaction, western blot, immunofluorescence, and/or immunohistochemistry. Compared with normal skin, mRNA expression of all ISGs in HPV-2 positive cutaneous warts was significantly decreased (*p* < 0.05). In comparison with empty vector transfection, *E7* transfection significantly down-regulated the mRNA and protein expressions of ISGs and type I IFN signaling pathway components, which were significantly up-regulated by *E7* siRNA transfection (*p* < 0.05). Interestingly, epigallocatechin-3-gallate (EGCG) pretreatment up-regulated the mRNA and protein expressions of ISGs and type I IFN signaling pathway components, which were significantly down-regulated by *E7* transfection (*p* < 0.05). Our results demonstrate that EGCG is a potential candidate for cutaneous wart prevention.

## 1. Introduction

Human papillomaviruses (HPV) are small, non-enveloped, double-stranded DNA viruses that infect cutaneous and mucosal epithelial tissues [1,2]. When infected with HPV, the innate host immunity becomes the first line of defense [3]. Following viral entry, HPV is recognized by pattern recognition receptors (PRRs) such as toll-like receptors (TLRs). The recognition of HPV infection by PRRs leads to the activation of a cascade of downstream signaling pathways [4]. However, HPV can evade immune surveillance to establish persistent infections. The evasion strategies of HPV are modulation of cytokine and chemokine expression, alteration of antigen presentation, and down-regulation of the interferon (IFN) pathway and adhesion molecules [5].

IFNs are components of the innate immune system that protect against viral infection through antiviral, anti-proliferative, and immune-stimulatory mechanisms [6]. IFNs act through their cell surface receptors to engage the Janus kinase (JAK)-signal transducer and activator of transcription (STAT) pathway [7]. Type I IFN signaling induces the transcription of interferon-stimulated genes (ISGs), whose protein products inhibit virus life cycles [8]. In the absence of detectable IFNs, constitutive ISG which is known to be critical for cellular susceptibility to viral infection is expressed. Constitutive ISG expression is mediated by the unphosphorylated-interferon stimulated gene factor-3 (U-ISGF-3) complex, which is formed by interferon regulatory factor (IRF)-9 and unphosphorylated STAT-1 and STAT-2 [9]. The U-ISGF-3 complex persists and is responsible for expression of a signature set of ISGs, many of them known antiviral proteins [10]. For example, interferon-stimulated gene 15 (ISG15) is one of the most highly induced ISGs, and modulates numerous cellular activities. Mx proteins are also highly induced by type I IFNs, and only human myxovirus-resistant protein A (MxA) has been shown to have antiviral activity. 2′-5′-oligoadenylate synthetase (OAS) proteins polymerizes ATP to 2′-5′-linked oligomers of adenylate, which activate Ribonuclease L to degrade viral RNA [11]. IFN response could be related to the regression of HPV infection, whereas HPV proteins interfere with the induction of the type I IFN signaling pathway. Microarray analyses have demonstrated that high-risk HPV proteins suppress the expression of a broad set of ISGs including MxA, OAS, and STAT-1 [12]. HPV-18 E6 has been reported to inhibit the JAK-STAT pathway by directly interacting with TYK2 [13]. HPV-16 E7 has also been shown to bind IRF-9 blocking the ISGF-3 complex translocation to the nucleus and ISGs expression [14]. Similarly, type 2 HPV (HPV-2) E7 has been reported to attenuate the expression of viperin (virus inhibitory protein, endoplasmic reticulum-associated, interferon-inducible) [15]. However, the effect of HPV-2 *E7* on antiviral ISGs is not yet clear.

Green tea contains various polyphenolic compounds known as catechins [16]. Epigallocatechin-3-gallate (EGCG) is one of the major components of these catechins, and has various bioactivities, including anti-oxidant, anti-viral, anti-proliferative, and immunomodulatory functions [17]. EGCG is currently being actively studied for its antiviral effect on diverse families of viruses such as HPV [18], human immunodeficiency virus (HIV) [19], and hepatitis C virus (HCV) [20] and for the prevention of carcinogenesis in various organs [21].

In this study, we investigated whether HPV-2 *E7* can inhibit ISGs expression by down-regulating the type I IFN signaling pathway. Furthermore, we examined the effect of EGCG on ISGs expression.

## 2. Results

### 2.1. The Expression of Interferon-Stimulated Genes Declined in HPV-2 Wart Samples

**Figure 1:**
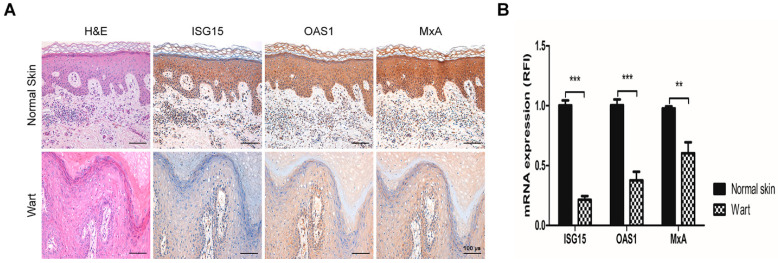
Expressions of interferon-stimulated genes in cutaneous wart samples. (**A**) Representatively image of hematoxylin and eosin staining (H&E) and immunohistochemical staining for interferon-stimulated gene 15 (ISG15), 2′-5′-oligoadenylate synthetase 1 (OAS1), and myxovirus-resistant protein A (MxA) in wart and normal skin samples. Original magnification = 200×, scale bar = 100 μm. (**B**) RT-qPCR analysis of interferon-stimulated genes in wart and normal skin samples. Number of wart samples = 6, number of normal skin samples = 5. The relative mRNA levels were normalized to that of *GAPDH*. The results are expressed as means ± SEM of three independent experiments. ** *p* < 0.01, *** *p* < 0.001 compared with normal skin.

We aimed to determine the ISGs expression levels in human tissues via immunohistochemistry and RT-qPCR. Immunohistochemistry results showed that the protein expressions of ISG15, OAS1, and MxA in the epidermis of HPV-2 positive warts were weaker than those of normal skin (Figure 1A). Likewise, the mRNA expressions of *ISG15*, *OAS1*, and *MxA* in wart samples were significantly decreased compared to normal skin (*p* < 0.01) (Figure 1B).

### 2.2. HPV-2 E7 Suppressed the Expression Of Interferon-Stimulated Genes by Down-Regulating the Type I Interferon Signaling pathway

**Figure 2:**
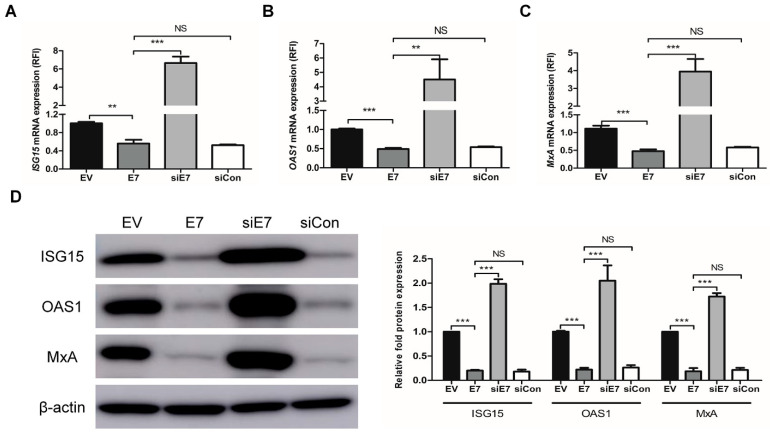
Effect of HPV-2 *E7* transfection on the expressions of interferon-stimulated genes. HaCaT cells were transfected with empty vector, *E7*, *E7* specific siRNA, and negative control siRNA (siCon). After transfection, mRNA expression levels of *ISG15* (**A**), *OAS1* (**B**), and *MxA* (**C**) were analyzed by RT-qPCR. The relative mRNA levels were normalized against levels of *GAPDH*. (**D**) Protein expression levels of ISG15, OAS1, and MxA were evaluated by western blot. The histogram shows quantitative representation of the protein levels of ISG15, OAS1, and MxA obtained from a densitometric analysis. β-actin served as a loading control for protein normalization. The data are shown as the means ± SEM of at least three independent experiments. ** *p* < 0.01, *** *p* < 0.001 compared with empty vector or *E7*. NS, non-significant.

**Figure 3:**
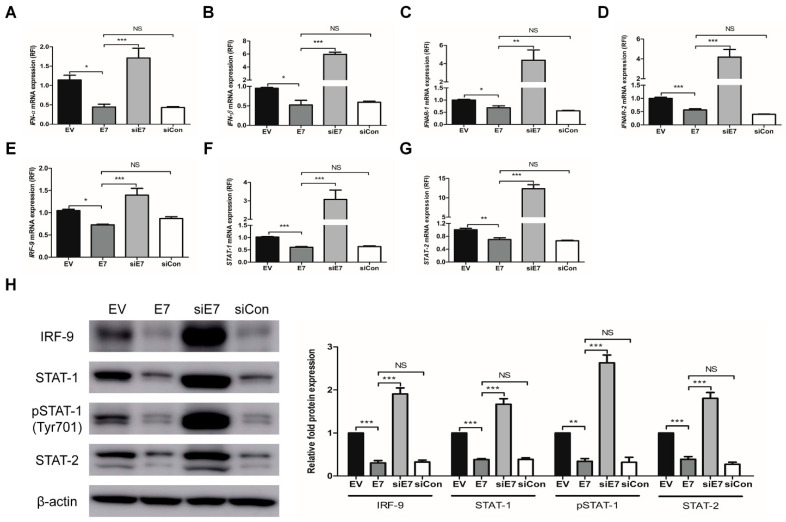
Effect of HPV-2 *E7* transfection on the expressions of type I interferon signaling pathway components. HaCaT cells were transfected with empty vector, *E7*, *E7* specific siRNA, and negative control siRNA. (**A**–**G**) The mRNA expression levels of type I interferon (IFN) signaling pathway components, including *IFN-α*, *-β* and their receptors (*IFNAR-1*, *2*), interferon regulatory factor-9 (*IRF-9*), signal transducer and activator of transcription (*STAT*)*-1,* and *STAT-2* were measured by RT-qPCR. The relative mRNA levels were normalized against levels of *GAPDH*. (**H**) The protein levels of IRF-9, STAT-1, STAT-2, and tyrosine phosphorylation STAT-1 were measured using western blot analysis. The histogram shows quantitative representation of the protein levels of unphosphorylated-ISGF-3 components obtained from a densitometric analysis. β-actin served as a loading control for protein normalization. The results are expressed as the mean ± SEM of three independent experiments. * *p* < 0.05, ** *p* < 0.01, *** *p* < 0.001 compared with empty vector or *E7*. NS, non-significant.

Next, we examined the effects of HPV-2 *E7* transfection on the expression of ISGs through the type I IFN signaling pathway using HaCaT cells. To further study the functional specificity of *E7* on the expression of ISGs, HaCaT cells were transfected with HPV-2 *E7*, *E7* specific siRNA (siE7), or negative control siRNA (siCon). *E7* transfection significantly down-regulated the mRNA and protein expressions of ISGs (including ISG15, OAS1, and MxA), which were significantly up-regulated by *E7* siRNA transfection (*p* < 0.01) (Figure 2). Likewise, *E7* transfection significantly down-regulated not only the mRNA expression of type I IFN signaling pathway components including *IFN-α*, *-β* and their receptors (*IFNAR-1*, *2*), *IRF-9*, *STAT-1* and *STAT-2*, but also the protein expression of the U-ISGF-3 components (including IRF-9, STAT-1, STAT-2, and tyrosine phosphorylated STAT-1), which were significantly up-regulated by *E7* siRNA transfection (*p* < 0.05) (Figure 3). Our results demonstrate that HPV-2 *E7* can suppress the expression of ISGs by the down-regulation of the type I IFN signaling pathway.

### 2.3. EGCG Pretreatment Inhibited HPV-2 E7 Transfection

**Figure 4:**
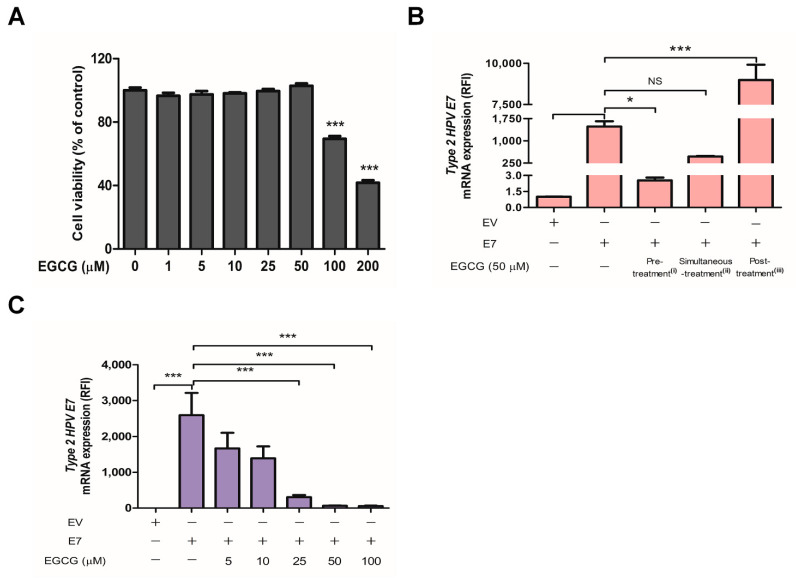
Effect of EGCG treatment against HPV-2 *E7* transfection. (**A**) EGCG toxicity in HaCaT cells. Cells were cultured in serum-free DMEM for 24 h, and then treated with EGCG at the indicated concentrations for 24 h. Cell viability was measured using MTT assay and expressed as a percentage of the value for untreated cells. The results are the mean ± SEM for three independent experiments. *** *p* < 0.001 compared with untreated cells. (**B**) HaCaT cells were incubated (i) with 50 μM EGCG for 2 h before HPV-2 *E7* transfection, then removed and performed transfection (Pre-treatment), (ii) with 50 μM EGCG for 24 h during transfection, then the EGCG and HPV-2 *E7* were removed (Simultaneous-treatment), or (iii) with 50 μM EGCG for 24 h after HPV-2 *E7* transfection and removal, then the EGCG was removed (Post-treatment). RNA was extracted from cells of the three groups and HPV-2 *E7* mRNA levels measured and expressed as a relative fold increase on the EGCG untreated controls. (**C**) HaCaT cells were treated with the indicated concentrations of EGCG for 2 h prior to *E7* transfection. After transfection, total RNA extracted from cells was subjected to RT-qPCR for mRNA levels of HPV-2 *E7*. The relative mRNA levels were normalized against levels of *GAPDH*. The results are the mean ± SEM for three independent experiments. * *p* < 0.05, *** *p* < 0.001 compared with empty vector or *E7*. NS, non-significant.

Before testing potential antiviral effects of epigallocatechin-3-gallate (EGCG) on HPV-2 *E7* transfection, we performed MTT assay to measure its toxicity for HaCaT cells at concentrations of 0–200 μM for 24 h. As shown in Figure 4A, cell toxicity of EGCG was seen at 100 and 200 μM, but not at 50 μM. To determine whether the optimal time of EGCG treatment to inhibit HPV-2 *E7* transfection, 50 μM EGCG was added before, during, and after transfection, respectively. The pretreatment with EGCG was significantly decreased the *E7* mRNA expression (*p* < 0.05), whereas addition of EGCG during or after *E7* transfection did not (Figure 4B). To further evaluate the effect of EGCG pre-treatment on *E7* transfection, we transfected with β-galactosidase reporter vector in cells treated with or without 50 μM EGCG. β-galactosidase reporter vector was used to monitor the transfection efficiency [22]. β-galactosidase activity was decreased by 63.5% in the EGCG-pretreated cells, but not in the untreated cells (Figure S1). These results supported that EGCG pretreatment block the *E7* expression by suppressing *E7* transfection efficiency. Additionally, EGCG pretreatment resulted in a dose-dependent decrease in the expression levels of HPV-2 *E7* mRNA (Figure 4C).

### 2.4. EGCG Pretreatment Sustained the Expression of Interferon-Stimulated Genes by Blocking HPV-2 E7 Expression

**Figure 5:**
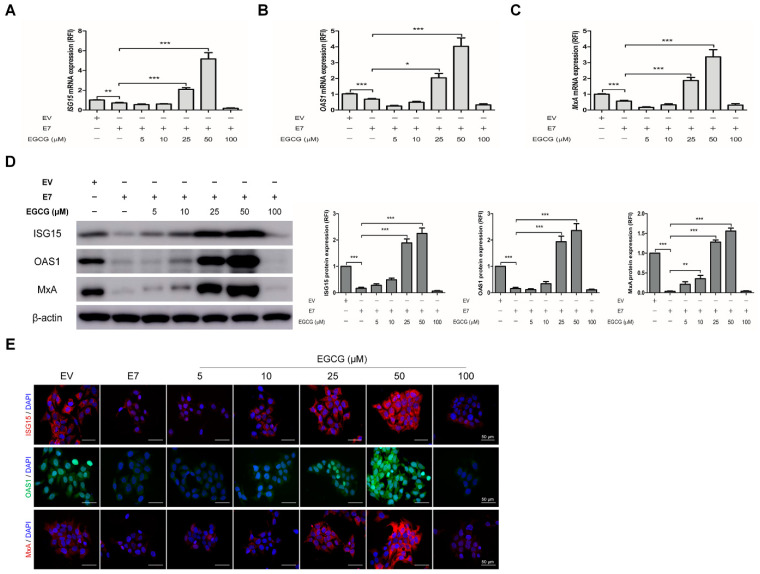
Effect of EGCG pretreatment on the expressions of interferon-stimulated genes in HPV-2 *E7* transfected cells. HaCaT cells were pretreated with EGCG at the indicated concentrations for 2 h before transfection, then the pretreated cells were transfected with empty vector or HPV-2 *E7*. (**A**–**C**) Total RNA extracted from cells after 24 h transfection was subjected to the RT-qPCR for the mRNA levels of *ISG15* (**A**), *OAS1* (**B**), and *MxA* (**C**). The data are expressed target mRNA levels relative fold to the empty vector. The relative mRNA levels were normalized against levels of *GAPDH*. (**D**) Cell lysates were collected for western blot to measure the ISG15, OAS1, and MxA expressions in cells pretreated with EGCG. The graph indicates quantitative representation of the protein levels of ISG15, OAS1, and MxA obtained from a densitometric analysis. β-actin served as a loading control for protein normalization. The results are expressed as the mean ± SEM of three independent experiments. * *p* < 0.05, ** *p* < 0.01, *** *p* < 0.001 compared with empty vector or *E7*. (**E**) Representatively image of immunofluorescent staining of ISG15 and MxA (red), OAS1 (green) in pretreated cell with EGCG. DAPI (blue) was used to counterstain nuclei. Images by fluorescent microscope with 400× magnification, scale bars = 50 μm.

**Figure 6:**
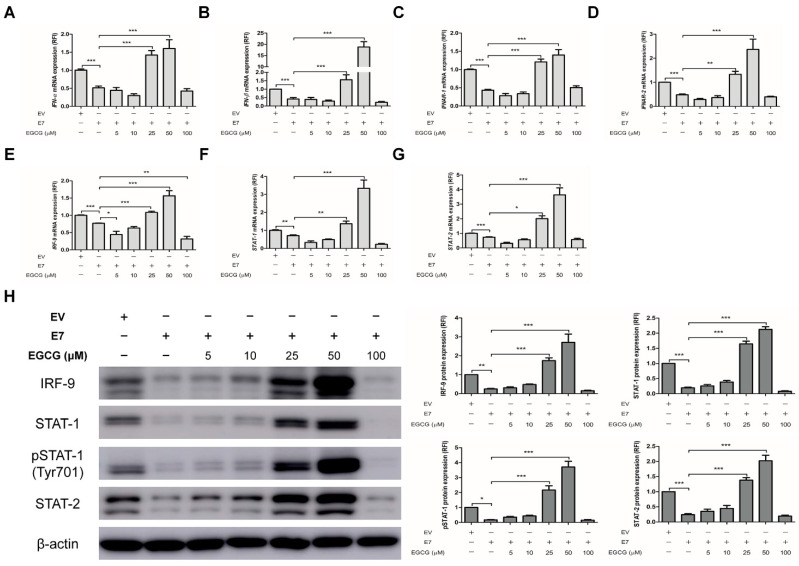
Effect of EGCG pretreatment on the expressions of type I interferon signaling pathway components in HPV-2 *E7* transfected cells. HaCaT cells were treated with EGCG at the indicated concentrations for 2 h prior to *E7* transfection. Following pretreatment with EGCG, the cells were transfected with empty vector or HPV-2 *E7*. (**A**–**G**) Total RNA extracted from cells after 24 h transfection was subjected to the RT-qPCR for the mRNA levels of *IFN-α* (**A**), *IFN-β* (**B**), *IFNAR-1* (**C**), *IFNAR-2* (**D**), *IRF-9* (**E**), *STAT-1* (**F**), and *STAT-2* (**G**). The relative mRNA levels were normalized against levels of *GAPDH*. (**H**) The protein levels of IRF-9, STAT-1, STAT-2, and tyrosine phosphorylation STAT-1 were measured using western blot analysis. The graph indicates quantitative representation of the protein levels of U-ISGF-3 components obtained from a densitometric analysis. β-actin served as a loading control for protein normalization. The results are expressed as the mean ± SEM of three independent experiments. * *p* < 0.05, ** *p* < 0.01, *** *p* < 0.001 compared with empty vector or *E7*.

Finally, we examined the effects of EGCG pretreatment on the expression of ISGs in *E7* transfected cells. EGCG pretreatment significantly up-regulated the mRNA expression of ISGs, which were down-regulated by *E7* transfection, at the concentration of 25 and 50 μM (*p* < 0.05), but not at 5, 10 and 100 μM (Figure 5A–C). A dose-dependent increase in ISGs protein expressions was also seen (Figure 5D). An immunofluorescence study showed that ISGs protein levels were increased in *E7* transfected cells pretreated with EGCG comparing with untreated *E7* transfected cells (Figure 5E). Unexpectedly, 100 μM of EGCG pretreatment had a decreasing tendency of the mRNA and protein expression of ISGs. Thus, we performed TUNEL (terminal deoxynucleotidyl transferase dUTP nick end labeling) assay to determine whether the effect of 100 μM of EGCG pretreatment was due to cell apoptosis. The proportion of TUNEL-positive cells was around 10% in EGCG-untreated cells and 50 μM EGCG-pretreated cells, which was increased to about 30% in 100 μM EGCG-pretreated cells (Figure S2). These results supported cell apoptosis by 100 μM of EGCG pretreatment. Likewise, EGCG pretreatment significantly up-regulated not only the mRNA expressions of type I IFN signaling pathway components including *IFN-α*, *-β* and their receptors (*IFNAR-1, 2*), *IRF-9*, *STAT-1* and *STAT-2*, but also the protein expression U-ISGF-3 components (including IRF-9, STAT-1, STAT-2, and tyrosine phosphorylated STAT-1), which were significantly down-regulated by *E7* transfection (*p* < 0.05) (Figure 6). Our results demonstrate that pretreatment with EGCG can prevent HPV-2 *E7* transfection, which can sustain the expression of ISGs and type I IFN signaling pathway components by blocking *E7* expression.

## 3. Discussion

In immunohistochemical staining, the expressions of ISGs such as ISG15, OAS1, and MxA commonly declined in HPV-2 wart samples. We also performed immunohistochemistry using cutaneous wart samples with different HPV types including type 1, type 1 and 27, and type 16. The expression of ISGs was also reduced in other types of wart samples as in HPV-2 (Figure S3). Likewise, Saadeh et al. showed that MxA expression decreased in uninflamed wart samples, in which host immune surveillance does not operate [23]. Based on these results, we suggest that the inhibition of ISGs expression allows HPV to maintain viral infection in human keratinocytes.

HPV has several mechanisms that evade the immune responses of host cells to sustain the infection, one of which interrupts the type I IFN signaling pathway. Among high-risk HPV proteins, E6 and E7 proteins can interfere with the expressions of type I IFNs in host cells [24], and can directly target components of the IFN pathway in order to inhibit their action, which leads to attenuation of ISGs [25]. Quite a few previous studies demonstrated that HPV-16 and -18 (high risk) E6 and E7 proteins can block transcriptional activity of the IRF family by binding to IRF-1, IRF-3, and/or IRF-9 [14,26,27]. In a previous report, we also found that HPV-2 E7 inhibited the expressions of IRF-1 and IRF-9 [15]. From complicated interactions not yet characterized, IRFs are deregulated by HPV proteins to diminish expression of IFN-α [12], IFN-β [26,27], and IFN-κ [28]. In addition to the E6 and E7 proteins, other HPV proteins can also interfere with the type I IFN signaling pathway. For example, the E1 proteins of HPV-11, 16, and 18 suppress up-regulation of IFN-β1, IFN-λ1 and ISG [29]. HPV-16 E2 decreases stimulator of interferon genes (STING) and IFN-κ expression, and HPV-18 E2 TAD-induced IFN-κ reduction leads to the down-modulation of the ISGs, such as interferon-induced protein with tetratricopeptide repeats (IFIT)1, IFIT3, Mx1, Mx2, OAS1, OAS2, and OAS3 [30]. HPV-16 E5 proteins suppress of IFN-κ transcription and IFN-κ-driven ISG expression and maintain the complete HPV-16 genome in keratinocytes [31].

In that previous study, we demonstrated that HPV-2 E7 attenuates the expression of viperin, one of the ISGs, but that E6 has little effect. Furthermore, in our preliminary study, normal human epidermal keratinocytes (NHEK) were transfected with either *E6* or *E7* to determine which is mainly involved in the suppression of ISGs expression. Compared to cells transfected with empty vector, the expression of ISGs in *E7* transfected cells decreased, but not in *E6* transfected cells. Accordingly, in this study we focused on whether HPV-2 *E7* can affect the expression of ISGs to evade antiviral immune responses, and we observed decreased ISGs expression even in HPV-2 *E7* transfected HaCaT cells. Our results demonstrate that HPV-2 *E7* directly targets the U-ISGF-3 complex resulting in the down-regulation of ISGs. Taken together, it is likely that the down-regulation of ISGs is important for the establishment and persistence of HPV-2 *E7* transfection.

EGCG, a catechin that has preventive and/or therapeutic effects in various diseases, exhibits broad and distinguished antiviral activity [32]. With regard to antiviral activities, EGCG usually targets the early stages of infection, such as attachment, entry, and membrane fusion [33]. In the present study, we found that pretreatment with EGCG inhibited HPV-2 *E7* transfection, but that posttreatment with EGCG did not. These contrasting results can be explained by differences in virus characteristics as well as EGCG treatment time.

In line with our findings, some studies have shown the effects of pretreatment with EGCG before viral infection on many viral species. Nance et al. reported that pre-incubation of EGCG for 24 h inhibits HIV-1 infectivity by preventing the attachment of HIV-1 glycoprotein 120 to the CD4 molecule [19]. Pretreatment with EGCG for 1 h prior to HCV stimulation significantly increased HCV induced IFN-λ 1, TLR3, retinoic acid-inducible gene (RIG)-I, and antiviral ISGs expression in both JFH-1-infected and -uninfected Huh7 cells [20]. When West Nile virus (WNV) was pretreated with EGCG for 1 h prior to infecting cell monolayers, EGCG significantly reduced virus yield and accumulation of intracellular viral RNA [34]. Pretreatment with EGCG for 3 h was also shown to increase antiviral mediators in the absence of influenza viral infection and also abrogate viral entry [35]. EGCG can inhibit the infectivity of a diverse group of enveloped and non-enveloped viruses by interrupting viral attachment to cell membrane receptors. The experiment was carried out by pretreating RNA or DNA virions with EGCG for 10 min or 1 h, and then infecting them into cell monolayers for 1 h [36]. In contrast, other studies have focused on the effects of posttreatment with EGCG after viral infection. In hepatitis B virus (HBV), EGCG treatment for 6 h after viral infection can inhibit the expression of HBV antigen and the synthesis of extracellular HBV DNA [37]. In HCV, EGCG treatment during inoculation inhibited HCV entry into hepatoma cell lines and primary human hepatocytes, by interrupting viral attachment to host cells [38]. Moreover, HIV also revealed that posttreatment with EGCG inhibits reverse transcriptase [39]. Based on our results, we suggest that pretreatment with EGCG is effective for preventing HPV infection, since EGCG should prevent the early stages of viral infection for immune escape.

In addition, we hypothesized that pretreatment with EGCG can sustain the expression of ISGs and type I IFN signaling pathway components because it blocks expression of HPV-2 *E7* by inhibiting *E7* transfection. Although from our results only, we have not been able to elucidate the exact mechanism for transcriptional inhibition of HPV *E7* by EGCG, we have found several possibilities from other experimental studies. First, Berger et al. reported that EGCG inhibits the activity of topoisomerase I, which plays crucial roles in DNA replication and transcription [40]. Second, Ahn et al. reported that EGCG downregulates RNA polymerase Ⅱ, which transcribes DNA as a precursor to mRNA in CaSki cells, human cervical cancer cell line [41]. Finally, Jacob et al. reported that EGCG treatment inhibits transcription of RNA polymerase Ⅲ, which transcribes many small structural RNA molecules involved in RNA processing and translation from both gene internal (tRNA) and external (U6 snRNA) promoters [42]. Based on those reports, it might be assumed that in our study EGCG inhibited the activity of several factors that play a crucial role in the HPV *E7* transcriptional mechanism.

In conclusion, our findings indicate that EGCG has the ability to bolster innate antiviral immunity against HPV-2, suggesting the potential application of EGCG as a preventive agent for cutaneous warts.

## 4. Materials and Methods

### 4.1. Tissue Samples

Cutaneous wart samples were taken from patients who had been pathologically diagnosed with common wart. We used six samples identified as HPV type 2 (HPV-2) through genotyping (Figure S4) performed by multiplex PCR [43]. As a control, normal skin samples were obtained from patients who underwent surgery in Department of Dermatology of Seoul St. Mary’s Hospital, the Catholic University of Korea. The protocol was approved by the Institutional Review Board of The Catholic University of Korea (MC20SASE0112) and performed according to the Helsinki Declaration.

### 4.2. Immunohistochemistry

Immunohistochemistry was performed on 5 mm thick sections obtained from 4% paraformaldehyde-fixed, paraffin wax-embedded tissue. Following dewaxing and rehydration steps, antigen retrieval was performed using citrate buffer. Sections were blocked with peroxidase blocking solution (Dako, Santa Clara, CA, USA) for 15 min. The sections were incubated with ISG15 (sc-166755, dilution of 1:100; Santa Cruz Biotechnology Inc., Dallas, TX, USA), OAS1 (ab82666, dilution of 1:100; Abcam, Cambridge, UK), and MxA (GTX110256, dilution of 1:500; GeneTex Inc. Irvine, CA, USA) overnight at 4 °C. After rinsing with phosphate-buffered saline (PBS), the sections were incubated with Dako REAL™ EnVision/HRP (Dako) at room temperature for 2 h, and were visualized with substrate-chromogen solution followed by counterstaining with Mayer’s hematoxylin (Dako). Stained tissue samples were observed using a Leica light microscope DMI 5000B (Leica, Wetzlar, Germany).

### 4.3. Cell Culture and Reagents

HaCaT cells, a naturally immortalized keratinocyte cell line derived from primary human epithelium, were obtained from the American Type Culture Collection (Manassas, VA, USA) and cultured in Dulbecco’s modified Eagle’s medium/high glucose (Gibco, Carlsbad, CA, USA) containing 1% penicillin/streptomycin (Gibco) and 10% fetal bovine serum (Gibco) in a humidified atmosphere containing 5% CO_2_ at 37 °C. Normal human epidermal keratinocytes (NHEK) from neonatal foreskin (Promocell, Heidelberg, Germany) were cultured in keratinocyte growth medium 2 with a supplement pack (Promocell) and 1% penicillin/streptomycin (Gibco). Epigallocatechin-3-gallate (EGCG) was purchased from Sigma-Aldrich (St. Louis, MO, USA). EGCG stock solution was prepared in sterile distilled water at 10 and 20 mM.

### 4.4. Cell Transfection

HaCaT cells were seeded (5 × 10^4^ cells/well) into six well plates and incubated overnight at 37 °C in 5% CO_2_. The cells were transfected with 2 μg of each DNA construct (pCMV::Δ and pCMV::2*E7*) and 50 nM HPV-2 *E7* specific siRNA (Invitrogen, Carlsbad, CA, USA) or negative control siRNA (Invitrogen) using TransIT-X2^®^ transfectant (Mirus, Madison, WI, USA) for 24 h according to the manufacturer’s instructions. DNA vectors were obtained from professor Soon Yong Choi (Hannam University, Daejeon, Korea).

### 4.5. Cell Viability Assay

HaCaT cells were seeded at a density of 1 × 10^4^ cells/well in 24 well plate. After 24 h, the medium was replaced with serum-free medium, then were treated with EGCG (1–200 μM) for 24 h, when cell viability was examined using MTT [3-(4,5-dimethylthiazol-2-yl)-2,5-diphenyl tetrazolium bromide (Sigma-Aldrich). MTT solution (100 µL, 5 mg/mL) was added to each well and incubated for 2 h at 37 °C. Then, the supernatant was aspirated, the MTT-formazan crystals by metabolically viable cells were dissolved in 400 µL of isopropanol (Merk Millipore, Burlington, MA, USA). The absorbance was measured at a wavelength of 540 nm using VersaMax microplate reader (Molecular Devices, San Jose, CA, USA).

### 4.6. EGCG Treatment

HaCaT cells were seeded at a density of 5 × 10^4^ per well in six well plates. After being cultured for 24 h, the medium was replaced with serum-free medium. After starvation, the cells were pre-treated with EGCG (5–100 μM) for 2 h prior to transfection. For the EGCG treatment optimal time experiment, the cells were treated with 50 μM EGCG (i) for 2 h before transfection, (ii) for 24 h after transfection, or (iii) for 24 h during transfection. The negative control cells were incubated with sterile distilled water used as EGCG solvent for equal to EGCG treatment time.

### 4.7. β-Galactosidase Assay

NHEK cells were seeded (1 × 10^5^ cells/well) into 6 well plates and were pretreated with 50 μM of EGCG for 2 h prior to transfection. Untreated cells were incubated with the same amount of sterile distilled water used as EGCG solvent for the same treatment time. Subsequently, the cells were co-transfected with empty vector or *E7* with pCMV-βgal-R1 vector using TransIT-X2^®^ transfectant (Mirus) for 24 h according to the manufacturer’s instructions. After transfection, cells were lysed with 5X reporter lysis buffer (Promega, Madison, WI, USA) and then used for assays. β-galactosidase assay was used to β-galactosidase enzyme assay system with reporter lysis buffer (E2000; Promega) according to the manufacturer’s instructions. The absorbance was measured at a wavelength of 420 nm using VersaMax microplate reader (Molecular Devices).

### 4.8. Quantitative Real-Time Polymerase Chain Reaction (RT-qPCR)

Total RNAs were isolated using Trizol^®^ reagent (Invitrogen) according to the manufacturer’s protocol. Equal amounts of RNA (1 μg) were reverse transcribed into cDNA using ReverTra Ace^®^ qPCR RT Master Mix with gDNA Remover (ToYoBo, Osaka, Japan) according to the manufacturer’s instructions. RT-qPCR was performed using a CFX96™ Real-Time PCR Detection System^®^ (Bio-Rad, Hercules, CA, USA) with MG 2X qPCR MasterMix Ⅱ (SYBR Green) (MGmed, Seoul, Korea) and specific primers. Conditions of the RT-qPCR reaction consisted of an initialization step for 10 s at 95 °C followed by two-step PCR for 45 cycles of 95 °C for 5 s (denaturation) and 58–60 °C for 30 s (annealing/extension). Results were normalized to the level of glyceraldehyde 3-phosphate dehydrogenase (*GAPDH*) gene expression. The analysis of relative gene expression data was conducted using the 2^−ΔΔCT^ method [44]. The primer sequences are listed in Table 1.

### 4.9. Western Blot

Cells were lysed in RIPA Lysis and Extraction Buffer (Thermo Scientific, Rockford, IL, USA) containing protease and phosphatase inhibitor cocktail (Thermo Scientific). Equal amounts (20 μg) of extracted protein were separated by 10–15% SDS-PAGE gel and transferred to polyvinylidene fluoride membranes (Merk Millipore). The membranes were blocked with 5% Bovine serum albumin (BSA)/Tris buffered saline with 0.1% of Tween 20 (TBS-T) for 1 h at room temperature and then incubated with indicated primary antibodies in 5% BSA/TBS-T overnight at 4 °C. The following primary antibodies were used in the procedures: mouse monoclonal anti-ISG15 (1:200, sc-166755), IRF-9 (1:100, sc-365893), and β-actin (1:2500, sc-47778; Santa Cruz Biotechnology), rabbit polyclonal anti-OAS1 (1:500, ab82666) and rabbit monoclonal anti-MxA (1:1000, ab207414; Abcam), rabbit monoclonal anti-STAT-1 (1:1000, #14994), Phospho-STAT1 (Tyr701) (1:1000, #9167), and STAT-2 (1:1000, #72604; Cell Signaling Technology, Danvers, MA, USA). The blot membranes were washed out in TBS-T four times and were incubated with horseradish peroxidase-conjugated Goat anti-Mouse or Rabbit IgG secondary antibodies (GTX213111-01 or GTX213110-01, GeneTex) for 2 h at room temperature. After four times washes with TBS-T, bands were visualized by ECL substrate (Thermo Scientific) with Amersham™ Imager 600 (GE Healthcare, Chicago, IL, USA). All observed band intensity was quantified using Image J software (NIH, Bethesda, MD, USA).

### 4.10. Immunofluorescence

For immunofluorescence analyses, HaCaT cells were seeded on eight well chamber slides at 2 × 10^3^ cells/well. After transfection, cells were fixed with 4% paraformaldehyde for 15 min, followed by PBS washes. Cells were then permeabilized at −20 °C for 10 min using ice-cold methanol and then blocked with 1% BSA in 1X PBS for 1 h. After blocking, cells were incubated with ISG15 (sc-166755, dilution of 1:50; Santa Cruz Biotechnology), OAS1 (ab82666, dilution of 1:100; Abcam), and MxA (ab207414, dilution of 1:250; Abcam) overnight at 4 °C. Following washes with PBS, cells were incubated with Alexa Fluor^®^ 594 Goat Anti-Mouse IgG (A-11005, Invitrogen), Alexa Fluor^®^ 488 Goat Anti-Rabbit IgG (A-11008, Invitrogen), and Alexa Fluor^®^ 594 Goat Anti-Rabbit IgG (A-11037, Invitrogen) secondary antibodies (each at a 1:400 dilution) for 2 h at room temperature in the dark. After four times washes with PBS, cover slips were mounted with Vectashield Antifade Mounting Medium (Vector Laboratories, Burlingame, CA, USA) containing 4′,6-diamidino-2-phenylindole (DAPI), to counterstain cellular nuclei. Images were obtained by a fluorescence microscope (Axiovert 200, Zeiss, Oberkochen, Germany).

### 4.11. TUNEL Assay

HaCaT cells were seeded in four well chamber slides at 1.5 × 10^4^ cells/well. After being cultured for 24 h, the medium was replaced with serum-free medium. After starvation, the cells were pretreated with EGCG at 50 and 100 μM or sterile distilled water as a control for 2 h before transfection. The pretreated cells were transfected with empty vector or HPV-2 *E7*. Cell apoptosis was examined with the In Situ Cell Death Detection Kit, Fluorescein (Roche, Mannheim, Germany) according to the manufacturer’s instructions. The cells were fixed, washed with PBS, and permeabilized with 0.1% Triton X-100 solution in 0.1% sodium citrate. After further washing in PBS, the cells were labelled for DNA breaks with terminal deoxynucleotidyl transferase (TdT) dUTP fluorescein nick end labeling (TUNEL, green fluorescence) reaction mixture. After three washes with PBS, cover slips were mounted with Vectashield Antifade Mounting Medium (Vector Laboratories) containing DAPI, to counterstain cellular nuclei. The apoptotic cells were observed under Axiovert 200 (Zeiss) and counted at magnification ×400. The proportion of apoptotic cells was calculated by counting the TUNEL-positive cells and by dividing this number by the total number of cells and then multiplying by 100%, in a minimum of five microscopic fields.

### 4.12. Statistical Analysis

All data were representative data from at least three independent experiments. Statistical analysis was performed via one-way analysis of variance (ANOVA) followed by Tukey’s multiple comparison test. Unpaired t-tests were used for comparisons of the two groups. All data are expressed as the mean ± standard error of the mean (SEM). All graphs were generated using GraphPad Prism 5 (La Jolla, CA, USA). Statistical significance was considered when *p* value was less than 0.05 (*, *p* < 0.05; **, *p* < 0.01; and ***, *p* < 0.001).

## Figures and Tables

**Table 1:** Primers sequences for RT-qPCR amplifications.

Target		Sequence (5′ – 3′)
*HPV-2 E7*	Forward	GACCTACATTGCGACGAGCA
	Reverse	GGACGGTTCTGCCACACTTA
*IFNAR-1*	Forward	TTGTGTGAAAGCCAGAGCAC
	Reverse	TCAAGAAGACTTTCGCAGCA
*IFNAR-2*	Forward	CACCAGAGTTTGAGATTGTTGG
	Reverse	GCTTGCTCATCACTGTGCTC
*IFN-α*	Forward	CTGAATGACTTGGAAGCCTG
	Reverse	ATTTCTGCTCTGACAACCTC
*IFN-β*	Forward	GCAGCAGTTCCAGAAGGAG
	Reverse	GCCAGGAGGTTCTCAACAAT
*IRF-9*	Forward	GTCCTGGGATGATACAGCTAAG
	Reverse	CAGGCGAGTCTTCCAGACAG
*STAT-1*	Forward	AGGAAAAGCAAGCGTAATCTTCA
	Reverse	TATTCCCCGACTGAGCCTGAT
*STAT-2*	Forward	GAGGAGAAGCAATGGGTCTTAG
	Reverse	GGTCCACAACCAACGAATAGA
*ISG15*	Forward	ACTCATCTTTGCCAGTACAGGAG
	Reverse	CAGCATCTTCACCGTCAGGTC
*OAS1*	Forward	CGGACCCTACAGGAAACTTG
	Reverse	TGATACCTCCTGGGATCGTC
*MxA*	Forward	ATCGGAATCTTGACGAAGCC
	Reverse	CCCTTCTTCAGGTGGAACAC
*GAPDH*	Forward	GAAGGTGAAGGTCGGAGTCAA
	Reverse	GCTCCTGGAAGATGGTGATG

## Data Availability

The data presented in this study are available in the article.

## Supplementary Materials

The following are available online at https://www.mdpi.com/1422-0067/22/5/2418/s1, Figure S1: Assessment of transfection efficiency upon EGCG pre-treatment. Figure S2: Measurement of TUNEL positive cells upon EGCG pre-treatment. Figure S3: Expression of interferon-stimulated genes in cutaneous warts with different HPV types. Figure S4: Result of multiplex PCR with cutaneous wart samples.

## Abbreviations

EGCG	Epigallocatechin-3-gallate
EV	Empty vector
HPV	Human Papillomavirus
IFN	Interferon
IRF-9	Interferon regulatory factors-9
ISG	Interferon-stimulated gene
ISGF-3	Interferon-stimulated gene factor-3
ISG15	Interferon-stimulated gene 15
MxA	Myxovirus-resistance protein A
OAS1	2’-5’-oligoadenylate synthetase 1
STAT	Signal Transducer and Activator of Transcription
