# Supplementary material for: Epigallocatechin-3-gallate Can Prevent Type 2 Human Papillomavirus E7 from Suppressing Interferon-Stimulated Genes
Source: Int J Mol Sci. 2021 Feb 28;22(5):2418. doi: 10.3390/ijms22052418 (PMC7957673; doi:10.3390/ijms22052418)
Supplement: Supplementary file 1 [file ijms-22-02418-s001.pdf]

Supplementary Figures

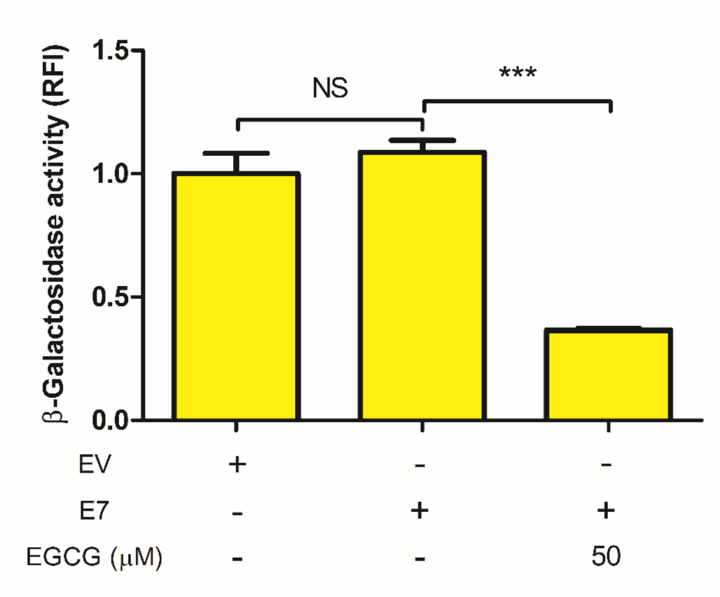

Figure S1. Assessment of transfection efficiency upon EGCG pre-treatment. Normal human epidermal keratinocytes (NHEK) cells were pretreated with 50 μM of EGCG for 2 h before transfection, then the cells were transfected with empty vector or *E7* together with pCMV-βgal-R1 as an internal control. 24 h after transfection, cells were harvested, and lysates were prepared to measure β-galactosidase activities. Values are presented as fold normalized activity relative to that of empty vector. The results are expressed as the mean ± SEM of at least three independent experiments. *** $p < 0.001$ compared with *E7*. NS, non-significant.

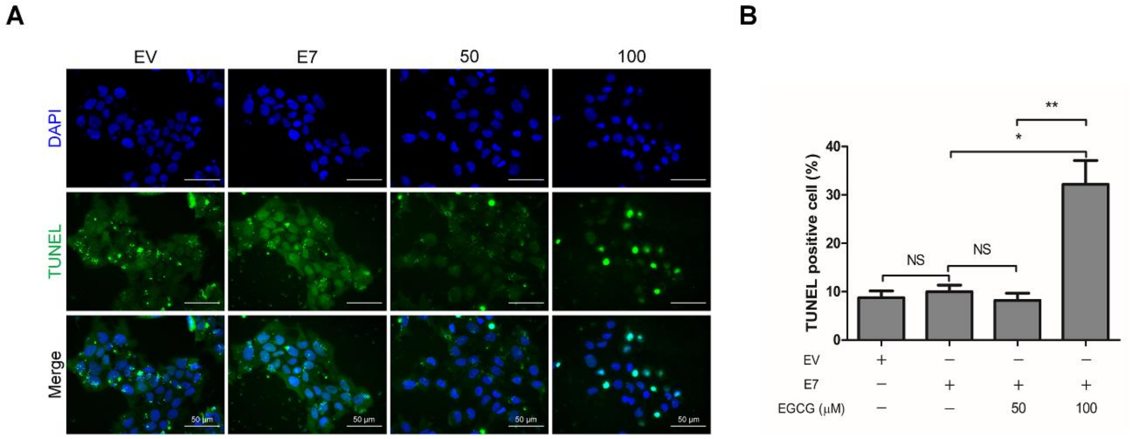

Figure S2. Measurement of TUNEL positive cells upon EGCG pre-treatment. HaCaT cells were pretreated with EGCG at the indicated concentrations for 2 h before transfection, then the pretreated cells were transfected with empty vector or HPV-2 *E7*. (**A**) The representative images of the TUNEL staining in pretreated cells with EGCG. TUNEL positive cells (green) were found to colocalize with nucleic marker DAPI (blue). Images by fluorescent microscope with x 400 magnification, scale bars = 50 μm. (**B**) The graph indicates quantification of the number of TUNEL positive cells. The results are expressed as the mean ± SEM of three independent experiments. * $p < 0.05$, and ** $p < 0.01$ compared with empty vector or *E7*. NS, non-significant; TUNEL, terminal deoxynucleotidyl transferase dUTP nick end labeling.

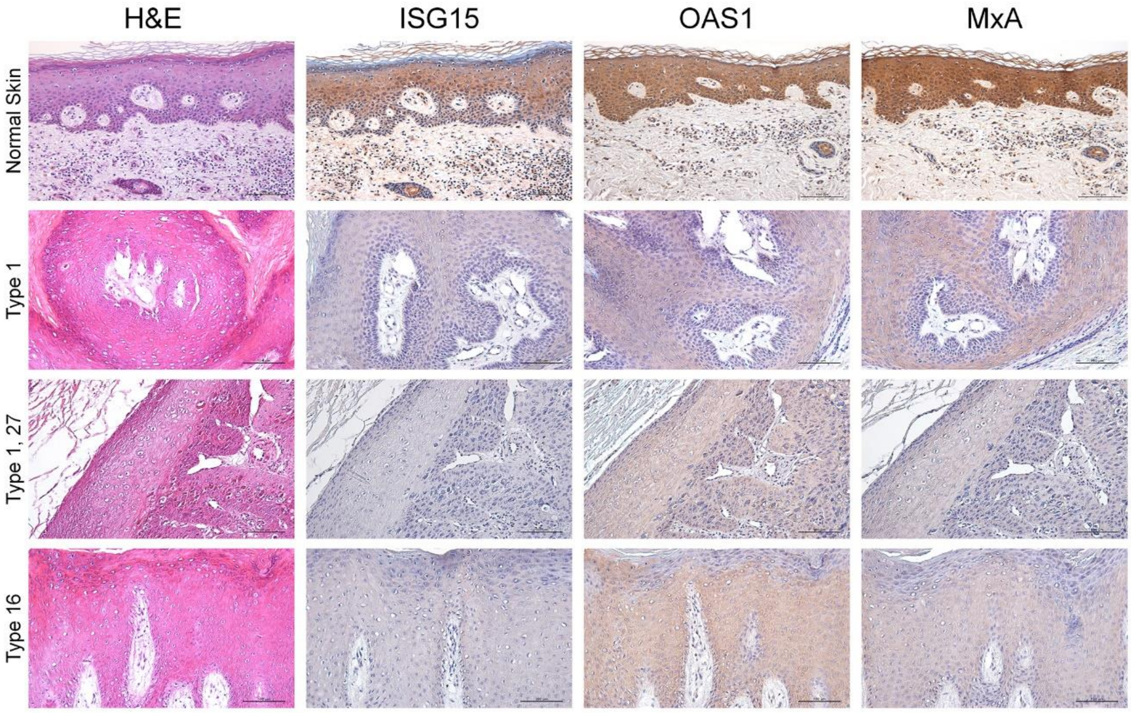

Figure S3. Expression of interferon-stimulated genes in cutaneous warts with different HPV types. Representatively image of hematoxylin and eosin (H&E) staining and immunohistochemical staining for ISG15, OAS1, and MxA in warts with different HPV types (type 1, type 1&27, and type 16) and normal skin samples. Original magnification = x 200, scale bars = 100 μm.

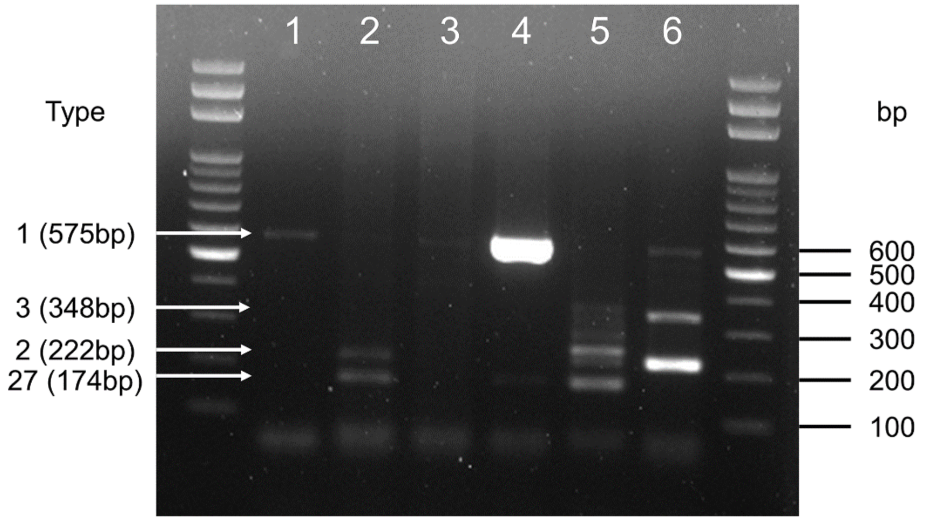

Figure S4. Result of multiplex PCR with cutaneous wart samples. HPV DNA isolated from cutaneous wart samples were amplified with HPV-1, -2, -3, -4, -27, and -57 types of different sizes primer sets. The DNA electrophoresis was carried out in 1.5% agarose gel to identify genotypes of PCR products. The bands of 575, 222, 348, and 174 bp of HPV-1, -2, -3, and -27 type were detected, respectively.
